# Differences in countermovement vertical jump force-time metrics between starting and non-starting professional male basketball players

**DOI:** 10.3389/fspor.2023.1327379

**Published:** 2023-12-08

**Authors:** Dimitrije Cabarkapa, Damjana V. Cabarkapa, Jelena Aleksic, Nicolas M. Philipp, Angeleau A. Scott, Quincy R. Johnson, Andrew C. Fry

**Affiliations:** ^1^ Jayhawk Athletic Performance Laboratory – Wu Tsai Human Performance Alliance, Department of Health, Sport and Exercise Sciences, University of Kansas, Lawrence, KS, United States; ^2^ Faculty of Sport and Physical Education, University of Belgrade, Belgrade, Serbia

**Keywords:** force, power, impulse, eccentric, concentric, sport, performance, monitoring

## Abstract

With force plates being widely implemented for neuromuscular performance assessment in sport-specific settings and various force-time metrics being able to differentiate athletes based on their performance capabilities, the purpose of the present study was to examine the differences in countermovement vertical jump (CVJ) characteristics between starting and non-starting professional male basketball players (e.g., ABA League). Twenty-three athletes (height = 199.2 ± 7.7 kg, body mass = 94.2 ± 8.2 kg, age = 23.8 ± 4.9 years) volunteered to participate in the present investigation. Upon completion of a standardized warm-up protocol, each athlete performed three maximal-effort CVJs without an arm swing while standing on a uni-axial force plate system sampling at 1,000 Hz. Independent *t*-tests were used to examine statistically significant differences (*p *< 0.05) in each force-time metric between starters (*n* = 10) and non-starters (*n* = 13). No significant differences in any of the CVJ force-time metrics of interest were observed between the two groups, during both the eccentric and concentric phases of the movement (i.e., impulse, duration, peak velocity, and mean and peak force and power). Moreover, starters and non-starters demonstrated similar performance on CVJ outcome (e.g., jump height) and strategy metrics (e.g., countermovement depth). Overall, these findings suggest that at the professional level of play, the ability to secure a spot in the starting lineup is not primarily determined by the players' CVJ performance characteristics.

## 1. Introduction

Basketball stands out as a fast-paced team sport characterized by its high-intensity intermittent nature which requires a combination of technical skills, strategic brilliance, and exceptional physical conditioning (1, 2). These requirements are particularly evident during live gameplay where basketball players typically engage in rapid changes in speed and direction and frequent jumping maneuvers, making neuromuscular performance an important cornerstone for success in this sport (3). Previous research has documented that neuromuscular performance characteristics of basketball players can differ based on age (4), sex (5, 6), and level of play (7–10). For example, top-tier basketball teams tend to have a better physiological capacity to sustain repetitive explosive actions over an extended period of time compared to lower-ranked teams (11). Similarly, national-level male basketball players demonstrated greater isometric midthigh-pull and countermovement vertical jump (CVJ) peak force when compared to their state-level age-matched counterparts (10). Moreover, these differences can also be position-specific (9, 12, 13). A considerably higher absolute leg muscle strength was found in centers than in guards (12), and lower reactive strength index, repeated reactive strength ability, and running vertical jump performance when compared to forwards and guards (13).

All of the aforementioned distinctions emphasize the complexity of the game of basketball and the need for in-depth player assessment to gain a better understanding of factors that contribute to success in this sport (14). With that in mind, one of the commonly implemented approaches used to evaluate the desired sport-specific physiological profile is by comparing athletes selected to play (i.e., starters) with those on the bench (i.e., non-starters) (15). So far, only a few studies have examined differences in various neuromuscular performance parameters between starters and non-starters in team sports such as soccer (16), volleyball (17), and rugby (15). Interestingly, the number of similar research reports pertaining to the game of basketball is even lower (18, 19). Overall, these studies tend to display superior neuromuscular performance characteristics in starters than non-starters, implying a potential influence of these findings on the selection process of the team's starting lineup.

As one of the commonly used testing modalities, the CVJ performed on force plates allows for non-invasive and time-efficient neuromuscular performance assessment in a sport-specific setting, mainly due to the simplicity of the testing protocol and a variety of force-time metrics that can be obtained with strong reliability (20–22). Depending on the purpose of the assessment, these force-time metrics are often used in return-to-play evaluation (23), monitoring fatigue-induced changes during practice and/or competition (24, 25), and assessing the athlete's overall neuromuscular performance capacities (26). Specifically, in the game of basketball, the CVJ has been used to monitor season-long neuromuscular performance changes (27) and distinguish players based on their jump strategy (28) and playing position (21). However, there is a lack of scientific literature that uses in-depth CVJ assessment to differentiate between starters and non-starters in the game of basketball, especially at the professional level of competition.

Thus, with force plates being widely implemented for neuromuscular performance assessment in the basketball-specific setting and various force-time metrics being able to differentiate athletes based on their performance capabilities, the purpose of the present study was to examine the differences in CVJ characteristics between starters and non-starters within a cohort of professional male basketball players.

## 2. Materials and methods

### 2.1. Participants

Twenty-three professional male basketball players ($\bar{x}$ ± SD; height = 199.2 ± 7.7 kg, body mass = 94.2 ± 8.2 kg, age = 23.8 ± 4.9 years) volunteered to participate in the present investigation. The players included in the starting lineup in more than 75% of the total games played during a full-season span were classified as starters (*n* = 10) and the rest of the players as non-starters (*n* = 13). The cohort of athletes encompassed two basketball teams competing at a similar level of play (e.g., ABA League) during a single competitive season. All athletes were free of musculoskeletal injuries and were granted permission to participate in team activities by their respective sports medicine staff. The testing procedures performed in this investigation were previously approved by the University's Institutional Review Board and all participants signed an informed consent document.

### 2.2. Procedures

The CVJ testing procedures were conducted during the middle of the regular season competitive period within the same time frame (i.e., 15:00–19:00 h) three days following the completion of the official game (e.g., the game was played on Sunday and CVJ testing was conducted on Wednesday) (21, 29). During this timeframe (Sunday–Wednesday) the athletes were not exposed to high-intensity fatiguing training sessions. Upon arrival to the gym for their regular team practice, all players completed a standardized 10-min warm-up procedure consisting of dynamic stretching exercises (e.g., walking lunges, squat-to-heel raise, A-skips, high knees, butt-kicks) administered by their respective strength and conditioning coaching staff. Then, each athlete stepped on a dual uni-axial force plate (ForceDecks Max, VALD Performance, Brisbane, Australia) and performed three maximal-effort CVJs without an arm swing (i.e., hands on the hips during the entire movement) with 10–15 s rest between each jump trial. If an athlete did not jump or land correctly, the CVJ trial was repeated. A strong verbal encouragement was provided throughout the testing procedures by research assistants, while instructing to focus on pushing the ground as hard and forcefully as possible (30). The force plate system sampling at 1,000 Hz was re-calibrated between each athlete and the mean value across three jumps was used for performance analysis purposes. Following the testing procedures, the players' age and height were obtained from the official team roster.

### 2.3. Variables

The dependent variables examined in the present investigation were based on the previously published research reports that demonstrated strong levels of validity and reliability for neuromuscular performance assessment (24, 31–33). The force-time metrics analyzed during the eccentric phase of the CVJ were: braking phase duration and impulse, eccentric duration, peak velocity, and mean and peak force and power. The force-time metrics analyzed during the concentric phase of the CVJ were: concentric duration, impulse, and peak and mean force and power. Alongside the detailed examination of the ground reaction force curve, the following CVJ metrics were derived: contraction time, jump height (i.e., impulse-momentum calculation), reactive strength index-modified (i.e., jump height divided by contraction time), and countermovement depth. The start of the contraction time was determined when the system mass was reduced by 20 N and ended at take-off (i.e., drop in vertical force below the 20 N threshold). The eccentric phase was defined as the phase with a negative center of mass velocity. As a subphase of the eccentric phase, the braking phase started at minimum force until the end of the eccentric phase. Impulse within both concentric and eccentric phases of CVJ were calculated as the area under the ground reaction force curve (21, 26, 27, 34). Additional information pertaining to data analysis software can be found at https://valdperformance.com/forcedecks/.

### 2.4. Statistical analysis

Shapiro–Wilk test and Q–Q plots corroborated that the assumption of normality was not violated. Independent *t*-tests were used to examine statistically significant differences in each CVJ force-time metric between starters (*n* = 10) and non-starters (*n* = 13). Due to the within-group sample size (*n* < 20), Hedge's *g* was used to calculate the magnitude of between-group differences (*g *= 0.2—small effect, *g *= 0.5—moderate effect, *g *= 0.8—large effect) (21, 35). Statistical significance was set *a priori* to *p *< 0.05. All statistical analyses were completed with SPSS (Version 26.0; IBM Corp., Armonk, NY, USA).

## 3. Results

Descriptive statistics, means and standard deviations ($\bar{x}$ ± SD), for each dependent variable are presented in Table 1 (anthropometric and comparison statistics) and Table 2 (CVJ force-time metrics and comparison statistics). No statistically significant differences were found between starters and non-starters in any force-time metrics examined in the present study (*p *> 0.05). In addition, the majority of the effect sizes were small to moderate in magnitude (*g *= 0.064–0.658).

**Table 1:** Anthropometric characteristics ($\bar{x}$ ± SD) and comparison statistics between starters and non-starters.

Variable [unit]	Starters	Non-starters	*p*-value	Effect size
Height [cm]	198.8 ± 7.7	199.6 ± 8.1	0.807	0.100
Body mass [kg]	97.1 ± 8.0	93.0 ± 7.9	0.134	0.516
Age [years]	25.6 ± 5.6	22.5 ± 3.9	0.170	0.658

**Table 2:** Countermovement vertical jump force-time metrics ($\bar{x}$ ± SD) and comparison statistics between starters and non-starters.

Variable [unit]	Starters	Non-starters	*p*-value	Effect size
Eccentric phase				
Braking phase duration [s]	0.293 ± 0.057	0.297 ± 0.066	0.884	0.064
Eccentric braking impulse [N·s]	66.1 ± 13.9	57.9 ± 16.5	0.111	0.531
Eccentric duration [s]	0.508 ± 0.095	0.495 ± 0.071	0.696	0.158
Eccentric peak velocity [m·s^−1^]	1.25 ± 0.20	1.17 ± 0.28	0.357	0.321
Eccentric peak force [N]	2,154.1 ± 352.9	2,128.9 ± 374.1	0.871	0.069
Eccentric mean force [N]	956.0 ± 79.1	914.2 ± 77.2	0.132	0.535
Eccentric peak power [W]	1,783.8 ± 580.3	1,514.7 ± 552.8	0.270	0.476
Eccentric mean power [W]	577.9 ± 113.9	517.5 ± 117.5	0.228	0.520
Concentric phase				
Concentric duration [s]	0.247 ± 0.044	0.265 ± 0.025	0.700	0.522
Concentric impulse [N·s]	265.8 ± 25.5	257.1 ± 23.5	0.411	0.357
Concentric peak velocity [m·s^−1^]	2.86 ± 0.26	2.91 ± 0.14	0.646	0.249
Concentric peak force [N]	2,573.6 ± 188.9	2,493.8 ± 423.5	0.586	0.232
Concentric mean force [N]	2,004.9 ± 141.5	1,972.6 ± 262.8	0.730	0.147
Concentric peak power [W]	5,669.0 ± 1,011.3	5,401.5 ± 702.1	0.462	0.315
Concentric mean power [W]	2,954.8 ± 297.5	2,979.2 ± 447.3	0.883	0.063
Other				
Contraction time [s]	0.751 ± 0.102	0.742 ± 0.103	0.649	0.088
Jump height [cm]	38.8 ± 7.4	40.0 ± 4.2	0.615	0.207
RSI-modified [ratio]	0.525 ± 0.080	0.559 ± 0.088	0.347	0.402
Countermovement depth [cm]	29.9 ± 4.1	28.8 ± 6.9	0.637	0.187

RSI, reactive strength index.

## 4. Discussion

To the best of our knowledge, this is the first study focused on examining differences in neuromuscular performance characteristics between starters and non-starters within a cohort of professional male basketball players. No significant differences were observed in any CVJ force-time metrics of interest between the two groups, during both the eccentric and concentric phases of the movement (e.g., impulse, duration, peak force, mean power). In addition, starters and non-starters demonstrated similar performance on CVJ outcome metrics (e.g., jump height) as well as strategy metrics (e.g., countermovement depth).

Previous literature has been primarily focused on examining anthropometric and physical performance characteristics of starters and non-starters in team sports such as volleyball, rugby, and soccer (15–17, 36, 37). The observed differences based on the players' ability to secure a spot in the starting lineup were not highly prominent, with the majority of performance parameters being comparable in magnitude (15, 17, 37), which is similar to the results obtained in the present investigation. For example, when studying a cohort of National Collegiate Athletic Association (NCAA) Division-I female volleyball players, Fry et al. (17) found no differences in the vertical jump height, isokinetic strength (i.e., quadriceps and hamstring peak torques), sprint (i.e., 9.1 m) and agility (i.e., *T*-test) performance, and isometric peak and mean force between the players included in the starting lineup and their substitutions. Similar observations were made by Gabbett et al. (15) when examining junior elite and sub-elite male rugby players. No significant differences were detected in vertical jump performance, sprint time and velocity (i.e., 10, 20, and 40 m), and change-of-direction ability (i.e., 505 test) between starters and non-starters at both levels of play (15). Also, it should be noted that the aforementioned research reports found no statistically significant differences in age, height, and body mass between the starters and non-starters (15, 17), which is identical to the results obtained in the present investigation. In addition, in a cross-sectional study conducted on state-level basketball players, Scanlan et al. (38) found no significant difference in change-of-direction speed between starters and non-starters. Combined, these findings suggest that the ability to secure the spot in the starting lineup on the same level of competition (e.g., junior, collegiate, professional) is not primarily determined by the players' anthropometric and physical performance characteristics. Although further research is warranted on this topic, it is likely that sport-specific skills (e.g., rebounding, shooting efficiency) and a player's ability to successfully execute offensive and defensive actions may have a greater impact in differentiating starters from non-starters in professional men's basketball (2).

Despite not being able to capture differences between starters and non-starters based on neuromuscular performance characteristics, the importance of strength and power development in team sport athletes should not be diminished. A recently published study revealed that greater values of lower-body strength and power were observed in basketball players competing at higher levels of play (i.e., collegiate vs. professional) (39). Further, when monitoring NCAA Division-I basketball players over a four-year competitive season span, Hoffman et al. (40) found a positive relationship between playing time and player's strength (i.e., one-repetition maximum), speed (i.e., 27 m sprint), and agility (i.e., *T*-test) performance. However, it is interesting to note that additional strength gains above the average values observed for a specific level of play do not seem to directly yield improvements in on-court performance (40). Thus, we can assume that both starters and non-starters examined in the present investigation already possessed adequate levels of strength and power. The values for CVJ force-time metrics observed during both eccentric and concentric phases of CVJ were similar in magnitude to the recently published research report focused on examining position-specific differences on a similar level of professional basketball play (21).

Although not reaching the level of statistical significance, moderate effect sizes observed within the eccentric phase of the CVJ should be noted (*g *= 0.520–0.535). When compared to non-starters, the players selected to be a part of the starting lineup tended to display slightly greater mean values in eccentric braking impulse, mean force, and mean power. In a similar investigation focused on examining a cohort of elite female professional basketball players, Spiteri et al. (41) found that eccentric strength was the strongest predictor on change-of-direction performance tests (i.e., 505 and *T*-test). Thus, considering the nature of the game of basketball and on-court competitive demands, we can assume that eccentric qualities are of critical importance for this specific group of athletes. In addition, another interesting observation pertaining to the results obtained in the present study is the moderate effect size difference in the age between the two groups (*g *= 0.685). Although further research is warranted on this topic, the starters being slightly older may imply that they had more basketball playing experience than non-starters (i.e., better technical-tactical understanding of the game through greater exposure to the sport) alongside displaying similar neuromuscular performance characteristics, which potentially allowed them to secure the spot in the starting lineup.

While providing practitioners with additional insight into neuromuscular performance characteristics of professional male basketball players, this study is not without limitations. The testing procedures were conducted at a single testing timepoint (i.e., in-season competitive period) for two teams competing on a similar level of play (e.g., ABA League). Alongside monitoring external load during practice and competition, implementing CVJ testing on a weekly or bi-weekly basis might provide additional insight into possible differences in force-time metrics between starting and non-starting players across a full season span. Also, further research needs to examine if the same findings apply to other competitive levels (e.g., amateur, collegiate) as well as if they are sex-specific.

In conclusion, the findings of the present study suggest that at the professional level of play, the ability to secure the spot in the starting lineup is not determined by the players' CVJ performance characteristics, but rather by other factors such as playing experience or their ability to proficiently execute sport-specific skills and offensive and defensive actions. These findings may help coaches, strength and conditioning practitioners, and sports scientists to obtain an additional insight into CVJ performance parameters of professional male basketball players as well as give direction and guidance when selecting assessments and training strategies targeted toward optimizing on-court basketball performance.

## Data Availability

The raw data supporting the conclusions of this article will be made available by the authors, without undue reservation.

## References

[1] Mancha-Triguero D García-Rubio J Antúnez A Ibáñez SJ. Physical and physiological profiles of aerobic and anaerobic capacities in young basketball players. Int J Environ Res Public Health. (2020) 17:1409. 10.3390/ijerph17041409 32098230 PMC7068281

[2] Cabarkapa D Deane MA Fry AC Jones GT Cabarkapa DV Philipp NM Game statistics that discriminate winning and losing at the NBA level of basketball competition. PLoS One. (2022) 17:e0273427. 10.1371/journal.pone.0273427 35984813 PMC9390892

[3] Latzel R Hoos O Stier S Kaufmann S Fresz V Reim D Energetic profile of the basketball exercise simulation test in junior elite players. Int J Sports Physiol Perform. (2018) 13:810–5. 10.1123/ijspp.2017-0174 29182413

[4] Pérez-Ifrán P Rial M Brini S Calleja-González J Del Rosso S Boullosa D Change of direction performance and its physical determinants among young basketball male players. J Hum Kinet. (2023) 85:23–34. 10.2478/hukin-2022-0107 36643832 PMC9808810

[5] Rice PE Goodman CL Capps CR Triplett NT Erickson TM McBride JM. Force- and power-time curve comparison during jumping between strength-matched male and female basketball players. Eur J Sport Sci. (2017) 17:286–93. 10.1080/17461391.2016.1236840 27691454

[6] Ziv G Lidor R. Physical attributes, physiological characteristics, on-court performances and nutritional strategies of female and male basketball players. Sports Med. (2009) 39:547–68. 10.2165/00007256-200939070-00003 19530751

[7] Ben Abdelkrim N Chaouachi A Chamari K Chtara M Castagna C. Positional role and competitive-level differences in elite-level men’s basketball players. J Strength Cond Res. (2010) 24:1346–55. 10.1519/JSC.0b013e3181cf7510 20393355

[8] Delextrat A Cohen D. Strength, power, speed, and agility of women basketball players according to playing position. J Strength Cond Res. (2009) 23:1974–81. 10.1519/JSC.0b013e3181b86a7e 19855320

[9] Ferioli D Rampinini E Bosio A La Torre A Azzolini M Coutts AJ. The physical profile of adult male basketball players: differences between competitive levels and playing positions. J Sport Sci. (2018) 36:2567–74. 10.1080/02640414.2018.1469241 29697296

[10] Williams MN Wen N Pyne DB Ferioli D Conte D Dalbo VJ Anthropometric and power-related attributes differ between competition levels in age-matched under-19-year-old male basketball players. Int J Sport Physiol Perf. (2022) 17:562–8. 10.1123/ijspp.2021-0079 35108672

[11] Ibáñez SJ Sampaio J Feu S Lorenzo A Gómez MA Ortega E. Basketball game-related statistics that discriminate between teams’ season-long success. Eur J Sport Sci. (2008) 8:369–72. 10.1080/17461390802261470

[12] Bradic A Bradic J Pasalic E Markovic G. Isokinetic leg strength profile of elite male basketball players. J Strength Cond Res. (2009) 23:1332–7. 10.1519/JSC.0b013e3181a0227e

[13] Pehar M Sekulic D Sisic N Spasic M Uljevic O Krolo A Evaluation of different jumping tests in defining position-specific and performance-level differences in high level basketball players. Biol Sport. (2017) 34:263–72. 10.5114/biolsport.2017.67122 29158620 PMC5676323

[14] Ostojic SM Mazic S Dikic N. Profiling in basketball: physical and physiological characteristics of elite players. J Strength Cond Res. (2006) 20:740–4. 10.1519/R-15944.1 17149984

[15] Gabbett T Kelly J Ralph S Driscoll D. Physiological and anthropometric characteristics of junior elite and sub-elite rugby league players, with special reference to starters and non-starters. J Sci Med Sport. (2009) 12:215–22. 10.1016/j.jsams.2007.06.008 18055259

[16] Yamaguchi S Inami T Yamashita D Nakamura M Kohtake N. Physical characteristics and performance of starters and non-starters in elite-level female soccer players in college: a case study of Japanese athletes. Football Sci. (2022) 19:49–58.

[17] Fry AC Kraemer WJ Weseman CA Conroy BP Gordon SE Hoffman JR The effects of an off-season strength and conditioning program on starters and non-starters in women’s intercollegiate volleyball. J Strength Cond Res. (1991) 5:174–81.

[18] Gonzalez AM Hoffman JR Scallin-Perez JR Stout JR Fragala MS. Performance changes in national collegiate athletic association division I women basketball players during a competitive season: starters vs. nonstarters. J Strength Cond Res. (2012) 26:3197–203. 10.1519/JSC.0b013e318273665d 22996019

[19] Gonzalez AM Hoffman JR Rogowski JP Burgos W Manalo E Weise K Performance changes in NBA basketball players vary in starters vs. nonstarters over a competitive season. J Strength Cond Res. (2013) 27:611–5. 10.1519/JSC.0b013e31825dd2d9 22648143

[20] Anicic Z Janicijevic D Knezevic OM Garcia-Ramos A Petrovic MR Cabarkapa D Assessment of countermovement jump: what should we report? Life. (2023) 13:190. 10.3390/life13010190 36676138 PMC9865236

[21] Cabarkapa D Philipp NM Cabarkapa DV Fry AC. Position-specific differences in countermovement vertical jump force-time metrics in professional male basketball players. Front Sports Act Liv. (2023) 5:1218234. 10.3389/fspor.2023.1218234 PMC10398786 37547821

[22] McMahon J Suchomel T Lake J Comfort P. An insight into the essential stages of the force-time curve in the countermovement jump. Strength Cond J. (2018) 40:96–106. 10.1519/SSC.0000000000000375

[23] Jordan MJ Aagaard P Herzog W. Lower limb asymmetry in mechanical muscle function: a comparison between ski racers with and without ACL reconstruction. Scand J Med Sci Sports. (2015) 25:301–9. 10.1111/sms.12314 25212216

[24] Cabarkapa D Cabarkapa DV Philipp NM Knezevic OM Mirkov DM Fry AC. Pre-post practice changes in countermovement vertical jump force-time metrics in professional male basketball players. J Strength Cond Res. (2023) 37:609–12. 10.1519/JSC.0000000000004608 37883409

[25] Gathercole RJ Stellingwerff T Sporer BC. Effect of acute fatigue and training adaptation on countermovement jump performance in elite snowboard cross athletes. J Strength Cond Res. (2015) 29:37–46. 10.1519/JSC.0000000000000622 25029001

[26] Merrigan JJ Stone JD Thompson AG Hornsby WG Hagen JA. Monitoring neuromuscular performance in military personnel. Int J Environ Res Public Health. (2020) 17:9147. 10.3390/ijerph17239147 33297554 PMC7730580

[27] Philipp NM Cabarkapa D Nijem RM Fry AC. Changes in countermovement jump force-time characteristics in elite male basketball players: a season-long analysis. PLoS One. (2023) 18:e0286581. 10.1371/journal.pone.0286581 37756277 PMC10529540

[28] Rauch J Leidersdorf E Reeves T Borkan L Elliott M Ugrinowitsch C. Different movement strategies in the countermovement jump amongst a large cohort of NBA players. Int J Environ Res Public Health. (2020) 17:6394. 10.3390/ijerph17176394 32887399 PMC7504515

[29] Philipp NM Cabarkapa D Eserhaut DA Cabarkapa DV Fry AC. Countermovement jump force-time metrics and maximal horizontal deceleration performance in professional male basketball players. J Appl Sports Sci. (2022) 2:11–27. 10.37393/JASS.2022.02.2

[30] Kershner AL Fry AC Cabarkapa D. Effect of internal vs. external focus of attention instructions on countermovement jump variables in NCAA division I student-athletes. J Strength Cond Res. (2019) 33:1467–73. 10.1519/JSC.0000000000003129 31125324

[31] Bishop C Turner A Jordan M Harry J Loturco I Lake J A framework to guide practitioners for selecting metrics during the countermovement and drop jump tests. Strength Cond J. (2022) 44:95–103. 10.1519/SSC.0000000000000677

[32] Merrigan JJ Strang A Eckerle J Mackowski N Hierholzer K Ray NT Countermovement jump force-time curve analyses: reliability and comparability across force plate systems. J Strength Cond Res. (2022):510–9. 10.1519/JSC.0000000000004586 37815253

[33] Heishman AD Daub BD Miller RM Freitas ED Frantz BA Bemben MG. Countermovement jump reliability performed with and without an arm swing in NCAA division 1 intercollegiate basketball players. J Strength Cond Res. (2020) 34:546–58. 10.1519/JSC.0000000000002812 30138237

[34] Philipp NM Cabarkapa D Eserhaut DA Yu D Fry AC. Repeat sprint fatigue and altered neuromuscular performance in recreationally trained basketball players. PLoS One. (2023) 18:e0288736. 10.1371/journal.pone.0288736 37459308 PMC10351699

[35] Hedges LV. Distribution theory for glass’s estimator of effect size and related estimators. J Educ Stat. (1981) 6:107–28. 10.3102/10769986006002107

[36] Marques MC Marinho DA. Physical parameters and performance values in starters and non-starters volleyball players: a brief research note. Motricidade. (2009) 5:7–11. 10.6063/motricidade.5(3).189

[37] Risso FG Jalilvand F Orjalo AJ Moreno MR Davis DL Birmingham-Babauta SA Physiological characteristics of projected starters and non-starters in the field positions from a division I women’s soccer team. Int J Exerc Sci. (2017) 10:568–79. 28674601 10.70252/ZOUT6574 PMC5466405

[38] Scanlan AT Tucker PS Dalbo VJ. The importance of open-and closed-skill agility for team selection of adult male basketball players. J Sport Med Phys Fit. (2015) 55(5):390–6. 26068324

[39] Cabarkapa D Fry AC Lane MT Hudy A Dietz PR Cain GJ The importance of lower body strength and power for future success in professional men’s basketball. Sports sci. Health. (2020) 10:10–6. 10.7251/SSH2001010C

[40] Hoffman JR Tenenbaum G Maresh CM Kraemer WJ. Relationship between athletic performance tests and playing time in elite college basketball players. J Strength Cond Res. (1996) 10:67–71.

[41] Spiteri T Nimphius S Hart NH Specos C Sheppard JM Newton RU. Contribution of strength characteristics to change of direction and agility performance in female basketball athletes. J Strength Cond Res. (2014) 28:2415–23. 10.1519/JSC.0000000000000547 24875426

